# Energy density and weight change in a long-term weight-loss trial

**DOI:** 10.1186/1479-5868-6-57

**Published:** 2009-08-14

**Authors:** Andrew Flood, Nathan Mitchell, Melanie Jaeb, Emily A Finch, Patricia S Laqua, Ericka M Welsh, Annie Hotop, Shelby L Langer, Rona L Levy, Robert W Jeffery

**Affiliations:** 1 University of Minnesota, Minneapolis, MN, USA; 2 Indiana University School of Medicine, Indianapolis, IN, USA; 3 University of Washington, Seattle, WA, USA

## Abstract

**Background:**

Health risks linked to obesity and the difficulty most have in achieving weight loss underscore the importance of identifying dietary factors that contribute to successful weight loss.

**Methods:**

This study examined the association between change in dietary energy density and weight loss over time. Subjects were 213 men and women with BMI of 30–39 kg/m^2 ^and without chronic illness enrolled in 2004 in a randomized trial evaluating behavioral treatments for long-term weight loss. Subjects completed a 62-item food frequency questionnaire at baseline and at 6, 12, and 18 months.

**Results:**

Pearson correlations between BMI and energy density (kcals/g of solid food) at baseline were not significantly different from zero (r = -0.02, p = 0.84). In a longitudinal analysis, change in energy density was strongly related to change in BMI. The estimated β for change in BMI (kg/m^2^) of those in the quartile representing greatest decrease in energy density at 18 months compared to those in the quartile with the least was -1.95 (p = 0.006). The association was especially strong in the first six months (estimated β = -1.43), the period with greatest weight loss (mean change in BMI = -2.50 kg/m^2 ^from 0–6 months *vs. *0.23 kg/m^2 ^from 12–18 months) and the greatest contrast with respect to change in energy density.

**Conclusion:**

Decreased energy density predicted weight loss in this 18 month weight loss study. These findings may have important implications for individual dietary advice and public health policies targeting weight control in the general population

## Background

National Health and Nutrition Examination Survey (NHANES) data indicate that in excess of 30% of Americans over the age 20 are obese (*i.e.*, have a body mass index or BMI in excess of 30 kg/m^2^)[1]. Furthermore, prevalence of obesity has shown rapid increase throughout the 1990's and has continued to rise unabated since then [1-3]. Given the well-established links between obesity and numerous chronic diseases (*e.g.*, heart disease, diabetes, hypertension, and many cancers) [4-6], identifying successful strategies for achieving weight loss would have significant public health impact.

Dietary energy density, or an individual's average daily energy intake divided by the grams of food consumed per day, is a factor that could help explain the broader trends in obesity prevalence and that could also serve as a potentially important point of focus in efforts to achieve weight loss. At the population level, dietary energy density is associated with energy intake and weight status in a limited number of cross-sectional studies [7-10]. At the individual level, higher energy density diets in feeding studies are associated with greater energy intake but not with a different volume of food consumed[11]. If changing to a diet comprised of low energy density foods results in fewer calories consumed, as these results suggest it should, then doing so should also result in a reduction in weight. Only a small number of longer-term studies have examined the question of changing energy density and its implications for weight loss [12-15]. In the only observational analyses from a study where energy density *per se *was investigated as a predictor of weight change, subjects with the greatest decrease in energy density achieved the greatest weight loss[16].

We conducted a longitudinal analysis of data collected from a randomized weight loss trial. It was our primary aim in these analyses to determine if a change in dietary energy density would result in weight loss over 18 months of follow-up, a follow-up period that extended beyond what has been reported in the small number of prior studies of energy density and weight loss. We had the secondary aim of analyzing the data at intermediate stages of follow up in order to explore the dynamics of the relationship between changes in energy density and weight loss and to provide insight into potential mechanistic explanations for the associations we observed.

## Methods

Subjects: Participants in the study were 100 men and 113 women recruited in 2004 and 2005 to participate in the Lose It Forever (LIFE) Study, a randomized clinical trial designed to compare the efficacy of prescribing different behavior combinations over time during therapy as a means of achieving and then maintaining weight loss.

Participants were recruited by general mass media advertising and specialized advertising targeting men and minorities. To be eligible for the study, subjects had to be at least 18 years of age, have a body mass index (BMI) between 30 and 39 kg/m^2^, be free from serious medical conditions or other consideration that would contraindicate treatment (i.e., participation in a weight loss intervention), agree to be randomized to either of two treatment groups, and provide their informed consent to participate. The study protocol was approved by the Institutional Review Boards at both the University of Minnesota and University of Washington.

Since the primary endpoint for this analysis was weight loss at 18 months of follow-up, we included only those subjects who had weight measurements at the 18 month clinic visit (see below) in the final analyses as well as in the cross-sectional analyses at baseline and in the analyses of intermediate time points. Doing so resulted in an analytic sample of 155 subjects (73% of those randomized). Including all subjects for the baseline cross-sectional analyses or including subjects with weight measures at the 6 or 12 month clinic visits but not the 18 month visit did not produce results materially different from those using only the subjects who had weight measures at 18 month completion point (data not shown).

LIFE Study Intervention: The present analysis uses the LIFE Study data in an observational manner, though the larger study used an experimental design as described here. Following enrollment, participants were randomized to one of two treatment groups. The treatment in both groups was conducted in small groups of 11 to 21 individuals at the Epidemiology Clinical Research Center at the University of Minnesota. The format of both treatment groups was comprised of a presentation by treatment staff of recommendations for changes in diet and physical activity behaviors necessary for successfully controlling weight, interactive discussion of these behavioral goals and strategies to achieve them, and homework assignments to be completed between sessions to reinforce learning.

Content for the Standard Behavioral Treatment (SBT) group was closely modeled after prior work of the investigators[17]and closely resembled the lifestyle intervention programs administered in recent successful clinical trials[18,19]. It was comprised of 26 weekly meetings over the first six months, bi-weekly meetings between months 6 and 12, and monthly meetings between months 12 and 18. Participants were given a calorie intake goal of 1,200, 1,500 or 2,000 kilocalories per day, depending on initial body weight, and were progressively advised to increase their level of moderate intensity physical activity (the primary exercise recommended was walking) to a total of one hour per day. The therapy in the Maintenance-Tailored Treatment (MTT) incorporated the same general diet and exercise goals but emphasized greater variety in both format and content. Energy density *per se *was not the focus of any of the weight loss units for either treatment arm. Encouragement to eat lower energy foods was a recurring theme, however, and individuals who act on this encouragement will often eat a lower energy density diet.

Outcome and Covariate Measures: The primary outcome was change in BMI. Study personnel used standardized procedures to measure subjects: Weight measurements were repeated, in light clothing without shoes, on an electronic scale during assessment sessions (in the morning, fasted) at baseline and at 6, 12, and 18 months of follow-up. Height was measured at the baseline assessment visit using a wall mounted stadiometer. LIFE Study personnel called participants prior to each six-month outcome assessment session to encourage attendance. In the event of a no-show at the assessment session, study personnel made multiple reschedule attempts.

Demographic characteristics of age, gender, and ethnicity were assessed at baseline. The Paffenbarger Activity Questionnaire, a well known assessment instrument with established validity and reliability [20-22], was used to estimate usual physical activity.

Dietary Assessment: Subjects completed the 62-item Block Food Frequency Questionnaire (FFQ) to assess usual intake in the previous 6 months. Subjects completed the questionnaires individually and in a self-administered manner (*i.e.*, without coaching from study personnel, although study personnel did review the FFQs for completeness accuracy when participants submitted them). Detailed descriptions of this FFQ and its validity have appeared elsewhere [23-25]. Software designed for this FFQ yielded estimates of daily intakes for total energy, macronutrients, and micronutrients, and grams of total foods and individual food items[25].

Statistical Analysis: We combined participants from both groups (MTT and SBT) into a single observational analysis. We used PROC CORR in the SAS statistical software package (version 8.2) to identify cross-sectional Pearson correlation coefficients among baseline values for BMI, energy consumed, and energy density of the diet. We used PROC GLM to generate beta estimates for change in BMI across quartiles of energy density change. We categorized subjects according to quartiles of change in energy density. In this way quartile 1 (Q1) contained subjects with a large decrease in energy density from baseline, and they had large negative values for change in energy density. Thus if we use Q4 (containing subjects with the least change in energy density – values close to 0) as the reference category, comparing Q1 to Q4 will compare those with large decreases in energy density to those with no change or even an increase in energy density. All beta estimates were derived from multivariable-adjusted models controlling for age, ethnicity, gender, change in physical activity level from baseline (based on Paffenbarger score), and treatment group (MTT *vs*. SBT) since treatment group was related to change in BMI (data not shown) and to change in energy density (Table 1). We report the results using three definitions of energy density for the cross-sectional correlations, but since associations were strongest for solid food, and since findings from prior studies in this area indicate that inclusion of beverages can diminish associations with outcome variables[26], we present in the tables only the results using a solid-food-only definition of energy density for all subsequent analyses. All statistical analyses took place in 2008.

**Table 1:** Baseline (unless otherwise indicated) characteristics of LIFE Study subjects according to quartile of change in energy density from baseline to 18 months of follow-up (all values are percents or means in units listed)

	**Quartile of Change in Energy Density from Baseline to 18 Months (N = 155)**			
	**1**	**2**	**3**	**4**
BMI (kg/m^2^)	34.8	35.2	35.4	33.8
Age (years)	50.4	50.1	49.4	50.6
Gender (% male)	41.0	52.6	62.5	47.4
Ethnicity (% white)	84.6	73.7	77.5	73.7
Treatment group (% randomized to MTT)	33.3	63.2	52.5	52.6
Smokers (current/former) (%)	43.6	36.8	47.5	31.6
More than HS education (%)	100	97.4	100	97.4
				
Energy density at baseline (kcals/g)	3.56	2.89	2.74	2.62
Energy density at 18 months (kcals/g)	2.26	2.30	2.48	2.85
Energy at baseline (kcals/day)	2003	1832	1691	1457
Quantity of food at baseline (g/day)	568	635	627	581
				
Physical activity at baseline (kcals/week)	771	1021	965	986
Physical activity at 18 months (kcals/week)	1848	2142	1736	1542
				
Vegetables (servings/day)^a^	1.65	2.18	2.63	2.73
Fruit (servings/day)^a^	0.41	0.49	0.71	0.78
Percent of Energy from Fat	49.2	47.5	46.1	44.6
Fiber (g/day)^a^	12.5	14.4	16.0	15.9

^a ^Energy adjusted so that values represent servings or grams per 2000 kcals.

## Results

Baseline characteristics for the LIFE study subjects, by quartile of change in energy density from baseline to 18 months, appear in Table 1. Interestingly, those with the greatest decrease in energy density from 0 to 18 months had the highest energy density at baseline, and across quartiles of change in energy density, the baseline energy density went down monotonically. By contrast, the quantity of food consumed was essentially the same across quartiles, and thus the energy consumption at baseline was lowest in the top quartile, where the energy density was also lowest. An equally interesting observation was that at 18 months, quartile 1, the quartile with the largest decrease in energy density, had the lowest mean energy density and across quartiles the energy density values increased, exactly the opposite of what we observed for baseline when quartile 1 had the highest mean energy density.

We observed no correlation between energy density and BMI at baseline in a cross sectional analysis (Table 2). This result did not change regardless of the definition we used for energy density (*i.e.*, defined in terms of all food, all food excluding non-dairy beverages, or solid food only). By contrast, we did observe a positive correlation between energy density and total energy consumed. This association was weak if we included any liquid foods in the definition of energy density, but was relatively strong (r = 0.30, p < 0.0001) if we considered only solid foods.

**Table 2:** Cross-sectional correlations among baseline measures of energy density, total energy intake, and BMI (N = 155)

	**Pearson Correlation Coefficients**		
	**Energy Density (kcals/g)**		
			
	**All Food**	**All Food – Excluding Non-dairy Beverages**	**Solid Food**

BMI (kg/m^2^)	0.08	-0.01	-0.02
	p = 0.35	p = 0.89	p = 0.84
			
Total Energy (kcals)	-0.01	0.09	0.30
	p = 0.87	p = 0.27	p < 0.0001

In a longitudinal analysis, the subjects who had the greatest decrease in energy density from baseline to 18 months had significantly decreased BMI compared to those who had the least change in energy density (Table 3). The multivariable-adjusted estimated β for Q1 (large decrease in energy density) *vs. *Q4 (no change or slight increase in energy density) was -1.95 kg/m^2 ^(p = 0.006), which is roughly equivalent to 5 or 6 kg Looking at different phases of follow-up, we observed that the estimated β was strongest in the first 6 months of the intervention period (β = -1.43, p < 0.0001 comparing Q1 to Q4) and grew noticeably smaller as time went forward such that by the 12 month to 18 month period the association was essentially null (β = -1.11, p = 0.01 for 6–12 months of follow-up; β = -0.31, p < 0.15 for 12–18 months of follow-up).

**Table 3:** Changes in energy density^a ^as predictors of changes in BMI^b ^and energy density

	**0–6 months**	**6–12 months**	**12–18 months**	**0–18 months**
**Estimated β for Δ BMI (kg/m^2^) resulting from Δ Energy Density (Q1 vs. Q4)^b^**	-1.43 p < 0.0001	-1.11 p = 0.01	-0.31 p = 0.15	-1.95 p = 0.006
				
**Mean Δ BMI**	-2.50 kg/m^2^	-1.20 kg/m^2^	0.23 kg/m^2^	-3.34 kg/m^2^
	(N = 153)	(N = 149)	(N = 151)	(N = 155)
				
**Mean Δ ED**				
**Q1 (N = 39)**	-1.39 kcal/g	0.11 kcal/g	-0.05 kcal/g	-1.31 kcal/g
**Q4 (N = 38)**	-0.05 kcal/g	-0.08 kcal/g	0.38 kcal/g	0.23 kcal/g

^a ^Defined in terms of solid food only.

^b ^Adjusted for age, gender, race and ethnicity, change in physical activity, and treatment group.

That the relationship between change in energy density and weight change would decline over time may at first appear puzzling, but it is important to note that the comparison that formed the basis of the β estimates (Q1 *vs. *Q4) during the different phases of follow-up represented much different levels of change in both the energy density and BMI variables during the different time periods. During the first six months of follow-up, Q1 represented a mean decrease in energy density of 1.39 kcal/g while Q4 represented a mean decrease of only 0.05 kcal/g. By contrast, in the 12–18 month period, Q1 represented a decrease in mean energy density of just 0.05 kcal/g while Q4 represented an increase of 0.38 kcal/g. Thus the difference between Q1 and Q4 was more than 3 times greater during the 0–6 month period than it was in the 12–18 month period. The absolute decrease in energy density in Q1 was more than 20 times greater during the early period of follow-up compared to the 12–18 month period.

In an effort to explore mechanistic explanations for the association between decreasing energy density and weight loss, we conducted a secondary analysis using a model that further controlled for total energy consumed. Contrary to expectations, doing so had no appreciable affect on the β estimates when comparing subjects with the largest reported decreases in energy density (Q1) to those who reported no change or a slight increase in energy density (Q4) (data not shown).

We also compared grams of food consumed and energy consumed at each clinic visit by quartile of energy density change between 0 and 18 months of follow-up (Table 4). Results from PROC MIXED indicated that there was no statistical difference in grams of food consumed over time in the four quartiles of 0–18 month change in energy density (p = 0.44), nor were the quartiles different in terms of grams consumed at baseline (p = 0.61). This was consistent with expectation and implied that if there was a change in energy density but no change in grams of food consumed, then those in the category representing greatest decrease in energy density must have been consuming fewer calories. This is in fact what we observed (Table 4). In Q1 of change in energy density, mean energy intake decreased from a baseline value of 2,003 kcals/day to 1,233 kcals per day at 18 months whereas in Q4, energy consumption remained essentially unchanged throughout the study (results from PROC MIXED indicating statistically significant interactions between time and quartile of 0–18 month change in energy density; p < 0.0001). Drawing on data from Table 1, it is clear that, on average, the subjects in Q1 were consuming more energy and had higher energy density diets at baseline than the subjects in Q4, but while the energy density in Q4 actually went up slightly during the study, it went dramatically down in Q1. Along with the lowered energy density (and constant volume of food consumed) came a dramatic decrease in energy consumed and significant weight loss. Meanwhile, in Q4 there were no changes (or even slight increases) in energy density and volume of food consumed, and therefore no change in energy intake, and no change in weight.

**Table 4:** Mean daily volume of food and energy consumed by quartile of baseline to 18 months change in energy density^a^

	**Grams of Food Consumed per Day (Std Dev)^b^**			
	**Baseline**	**6 mo.**	**12 mo.**	**18 mo.**
**0–18 Month Δ Energy Density**				
**Q1 – decrease (N = 39)**	568 (277)	544 (190)	498 (184)	553 (226)
**Q2 (N = 38)**	635 (232)	577 (279)	563 (222)	528 (151)
**Q3 (N = 40)**	627 (204)	519 (169)	576 (231)	539 (199)
**Q4 – increase (N = 38)**	581 (232)	513 (272)	516 (216)	499 (250)

	**Kcals of Energy per Day (Std Dev)^c^**			
	**Baseline**	**6 mo.**	**12 mo.**	**18 mo.**

**0–18 Month Δ Energy Density**				
**Q1 – decrease (N = 39)**	2,003 (1131)	1,184 (492)	1,122 (468)	1,233 (558)
**Q2 (N = 38)**	1,831 (737)	1,278 (533)	1,219 (505)	1,202 (380)
**Q3 (N = 40)**	1,692 (602)	1,240 (455)	1,323 (641)	1,346 (721)
**Q4 – increase (N = 38)**	1,457 (530)	1,261 (759)	1,234 (572)	1,367 (640)

^a ^Defined in terms of solid food only.

^b ^Results from PROC MIXED indicate no differences in volume of food consumed over time by quartile of change in energy density from 0 to 18 months and no difference in grams of food consumed across quartiles at baseline.

^c ^Results from PROC MIXED indicate significant differences (p < 0.0001) for change in kcals consumed over time by quartile of change in energy density from 0 to 18 months and a significant difference (p < 0.0001) in kcals consumed across quartiles at baseline.

## Discussion

In this 18 month weight loss intervention study, we observed that subjects with the greatest degree of change in energy density did not change the amount (*i.e.*, weight) of food they consumed but did have notable changes in energy intake. These observations are consistent with what Rolls and colleagues have observed in short-term feeding studies where study subjects consuming diets differing in energy density ate a constant volume of food but the low energy-density diet subjects consumed significantly fewer calories[10,11,27,28]. After controlling for changes in physical activity during the intervention and for other potential confounders, we further found that the changes we observed in energy density (and in mean daily energy consumption) were associated with significant changes in weight.

Some of our findings were somewhat unexpected, however. For example, despite prior studies consistently showing an association between energy density of the diet and BMI [7-10], we found there was no correlation at baseline between energy density and BMI among LIFE Study subjects. A key difference in our study may be the range of values for BMI among study subjects at enrollment. By design, everyone in the LIFE Study was obese (*i.e.*, had a BMI in excess of 30 kg/m^2^), but no one had a BMI above 39 kg/m^2^. By restricting the range of values for BMI in this way, we may have obscured the cross-sectional association most previous investigators have observed.

A second unexpected finding was that the association between change in energy density and change in weight or BMI was different at different phases of follow-up. During periods of active weight loss, that weight loss was strongly correlated with decreased energy density, but later in follow-up, the association was markedly attenuated. Our evaluation of the magnitude of change in energy density during each of these time periods showed that large changes in energy density, both in absolute magnitude (*i.e.*, -1.39 kcal/g for those in the quartile with the greatest decline in energy density from 0–6 months compared to -0.05 kcal/g for those showing the greatest decline in energy density from 12–18 months) and in relative difference (1.34 kcal/g difference in mean change in energy density for Q1 *vs. *Q4 from 0–6 months compared to 0.43 kcal/g from 12–18 months) suggest that this apparently changing association may simply be a function of the greater range in exposure levels early in the follow-up period compared to later.

The underlying theory as to why decreasing energy density would result in weight loss or lower BMI is that food volume is an important determinant of food intake[11]. Decreased energy density in the context of constant volume of food consumption would necessarily involve less energy intake, just as we observed in the LIFE Study. If we control for changes in physical activity, then the lower energy intake should result in weight loss meaning the energy density effect is mediated by total energy consumption. And yet, in secondary analyses, when we adjusted for total energy, the estimated β's were largely unaffected (data not shown). If energy were truly the mechanism by which changes in energy density impacted BMI, then controlling for energy should have eliminated the effect of changing energy density. That it did not could potentially be explained as evidence that the association we saw was the result of confounding by some unmeasured or imperfectly measured factor.

Rolls and colleagues found results consistent with ours in a 12 month study of 200 overweight and obese individuals. In this study all subjects were on a calorie restricted diet, but after 1 year, subjects consuming a low energy density soup twice a day as compared to subjects eating a high energy density snack with the same energy content as the soup had significantly greater weight loss[13].

That the association of energy density with weight loss and energy intake was strongest if we defined it only in terms of solid food consumed (*i.e.*, if we excluded liquid food), is worth noting. This result suggest that the variation in energy consumption that comes from changes in energy density is primarily a function of solid foods, which should not be surprising as liquid foods as a rule are low in energy density[29]. Furthermore, Rolls and colleagues have found that varying the amount of drinking water with meals did not affect volume of food consumption[30] suggesting the effects of changes in energy density would be concentrated at the level of solid foods. As such, it is clear that substituting low energy-density solid foods in place of high energy density solid foods will impact energy intake, but the effects of liquid foods are much less obvious. Our results are consistent with these previous observations.

An interesting secondary observation in this study was the poor weight loss outcome in subjects starting with low energy density diets at baseline. There are several possible explanations for this result. The first is simply that subjects eating high energy density diets had substantial room for improvement on that front, and when they did reduce the energy density of their diets, their energy intake dropped in turn. For subjects with low energy density at baseline, however, no additional improvement of this type was possible. For the latter group of subjects, reducing energy intake could not happen through changes in energy density of the diet, and alternative strategies (*i.e.*, portion control) would be required for effective weight loss. Alternatively, the low energy density diets reported in the quartile showing the least change in energy density over the course of the study might be erroneous and as such reflect poor dietary self awareness. Given that all subjects had starting BMIs of between 30 and 39 kg/m^2 ^and thus had similar degrees of energy imbalance, it is perhaps not unreasonable to consider that those reporting the lowest energy density diets were in fact underestimating the amount of high energy density foods consumed relative to the rest of the study population. Thus *reporting *a low energy density diet at baseline may be an indicator of poor behavioral compliance (and hence little improvement in energy balance) among subjects who do not recognize the energy density of their diets at the outset. These explanations are strictly hypothetical, however, and require further investigation in future studies before any conclusions are possible.

The use of a food frequency questionnaire to assess energy density of diet might be a point of criticism for this study as most investigations of energy density have been short term feeding studies with tightly regulated (and measured) food intakes. There is no question that food frequency questionnaires measure diet with error, so much so that some have suggested dispensing with them altogether[31]. But this criticism is aimed primarily at the failure of studies using FFQs, due to the substantial random error inherent in those instruments, to find positive associations. In the case of the present analysis, we found that greater reduction in energy density resulted in greater weight loss, and dismissing that result on the grounds that the FFQ is inadequate to assess diet properly requires a belief that there is not simply random error but rather reporting bias as a function of success in weight loss. In other words, there would need to have been systematic *over *reporting of "good" habits (eating low energy density foods) in those who were successful at weight loss. While we cannot exclude this possibility, the reverse scenario (social desirability-motivated over-reporting of "good" behaviors in those who failed to lose weight) seems much more likely. That we found a positive result despite the limitations of the FFQ is notable.

A final limitation of the study may be in the demographic makeup of the study population. Results from this mostly white, generally well-educated population (in excess of 97% had more than a high school education) may not be generalizable to the broader public. Certainly, it is reasonable to consider if achieving these changes in dietary energy density would be similarly practical in different populations, but it is not immediately clear how change in energy density *per se *could have different effects in populations with different ethnic or educational profiles. Nonetheless, some caution is warranted in interpreting these results.

## Conclusion

In summary, decreases in energy density predicted weight loss in an 18 month observational analysis. Changes in energy density predicted weight loss most strongly in the first 6 months of follow-up. Diminished predictive value of energy density later in follow-up may be due to smaller changes in energy density during this period making for a less sensitive analysis. Volume of food consumed did not change with decreasing energy density suggesting energy intake went down, but curiously, controlling for energy intake did not eliminate the effect of changing energy density on weight loss.

## Competing interests

The authors declare that they have no competing interests.

## Authors' contributions

AF participated in the design of the study, designed the analysis, interpreted the data, and drafted the manuscript. NM performed the data analysis and assisted in drafting the manuscript. MJ and EAF assisted in the design of the study and supervised the operations of the weight loss intervention activities. SLL, PSL, EMW, and AH directed the intervention activities. RLL, and RWJ designed the study, provided overall study management, and assisted in the drafting of the manuscript. All authors read and approved the final manuscript.
